# *ICAM‐1* and *MKL‐1* polymorphisms impose considerable impacts on coronary heart disease occurrence

**DOI:** 10.1111/jcmm.15645

**Published:** 2020-08-07

**Authors:** Cungang Wu, Chao Huang

**Affiliations:** ^1^ Department of Ultrasonography The First Affiliated Hospital of Jinzhou Medical University Jinzhou China; ^2^ Department of Stomatology The First Affiliated Hospital of Jinzhou Medical University Jinzhou China

**Keywords:** coronary heart disease, *ICAM‐1*, *MKL‐1*, polymorphism

## Abstract

This study was aimed to explore the correlation of intercellular adhesion molecule‐1 (*ICAM‐1*) K469E and megakaryoblastic leukaemia factor‐1 (*MKL‐1*) −184C/T polymorphisms with the susceptibility to coronary heart disease (CHD) in the Chinese Han population. 100 CHD patients and 91 healthy people that had no blood connection with each other were enrolled in this case‐control study. *ICAM‐1* and *MKL‐1* polymorphisms were genotyped by polymerase chain reaction‐restriction fragment length polymorphism (PCR‐RFLP) approach. Multiple logistic regression was used to analyse the correlation between polymorphisms of *ICAM‐1* and *MKL‐1* and CHD susceptibility. Differences of genotype and allele frequencies of the two SNPs between case and control groups were analysed by chi‐square test. Odds ratios (ORs) and 95% confidence intervals (CIs) were indicated relative susceptibility of CHD. The distributions of *ICAM‐1* and *MKL‐1* polymorphisms in each group conformed to Hardy‐Weinberg equilibrium (HWE). After adjusting for traditional risk factors, the TT genotype frequency of *MKL‐1* −184C/T polymorphism was found significantly higher in case group than in control group (*P* < .05). Meanwhile, T allele frequency increased in case group compared with control group, and the differences had statistical significance (*P* = .04, OR = 2.34, 95% CI = 1.34‐5.26). Logistic regression analysis in this study proved that smoking, hypertension, diabetes and triglyceride (TG) were all risk factors for CHD *ICAM‐1* K469E polymorphism has no association with the onset of CHD. But *MKL‐1* −184C/T polymorphism is associated with the risk of CHD and T allele might be a susceptibility factor for CHD.

## 1 INTRODUCTION

Coronary heart disease (CHD) is due to myocardial ischaemia, hypoxia or necrosis, which is caused by coronary morphological changes of stenosis or obstruction, or coronary functional change of vascular spasm based on atherosclerosis.^1^, ^2^, ^3^ CHD is the most common and most frequent cardiovascular disease (CVD).^4^ At present, it has become one of the main diseases threatening the health of the people around the world, and its morbidity and mortality are increasing in China in recent decades.^5^, ^6^

Coronary heart disease is a complex disease. Its specific pathogenesis is not yet fully understood. But most scholars believe that the onset of the disease is closely related to environmental and genetic factors.^7^ Studies have shown that intercellular adhesion molecule‐1 (*ICAM‐1*), as an important inflammatory mediator and credible inflammatory marker, is a momentous factor for inducing local inflammation and thrombosis and plays a vital role in the pathological process of ischaemia cardio‐cerebrovascular diseases.^8^, ^9^, ^10^ The increased *ICAM‐1* levels are proved to be relevant to the risk of CHD.^11^, ^12^ In addition, megakaryoblastic leukaemia factor‐1 (*MKL‐1*), also known as myocardin‐related transcription factor‐A (*MRTF‐A*), is a member of the family of myocardin‐related transcription factors (MRTFs). The literature has reported that *MKL‐1* might influence the formation and maintaining process of the cardiovascular system by regulating the abnormal proliferation of smooth muscle cells ^13^, ^14^, ^15^ and take part in the occurrence and development process of CHD via RhoA and transforming growth factor‐beta1 (TGF‐β)–dependent channels.^16^ However, the domestic and foreign research results about the relationship between polymorphisms of *ICAM‐1* and *MKL‐1* and CHD were inconclusive and contradictory.

Therefore, in the present study, we discussed the correlation of *ICAM‐1* K469E and *MKL‐1* −184C/T polymorphisms with CHD susceptibility, and explored the effect of other environmental exposures on the onset of CHD.

## 2 MATERIALS AND METHODS

### 2.1 Cases and controls

The case group of the study enrolled 100 CHD patients including 58 males and 42 females. The mean age of the patients, who were diagnosed in Cardiology department of The First Affiliated Hospital of Jinzhou Medical University, was 59.4 ± 7.33 years old. Coronary angiography examinations of every patient showed that more than one main coronary artery of the cases had 50% or higher level of stenosis. Patients suffered from cardiomyopathy, haemorrhagic diseases, renal failure and malignant tumours were eliminated. The control group recruited 91 homochronous healthy persons (53 males and 38 females) from the physical examination centre of the same hospital, with a mean age of 60.3 ± 6.79 years old. The controls had no malignant tumours and immune inflammatory diseases, and they were proved without CHD history by electrocardiograph (ECG) examinations. All of the subjects were Chinese Han population, and they had no blood relationship with each other. Informed consents were obtained from each participant. And the study was approved by the Ethics Committee of The First Affiliated Hospital of Jinzhou Medical University.

### 2.2 Blood collection and DNA extraction

We collected 2 mL fasting venous blood of each subject and put the blood into PCR tubes, anticoagulated by EDTA. Genome DNA was extracted from 300 μL whole blood by Biospin Whole Blood Genomic DNA Extraction Kit (Sangon Company) and then stored at −70°C.

### 2.3 PCR amplifications and genetic typing assay

The genotypes of *ICAM‐1* K469E and *MKL‐1* −184C/T polymorphisms were examined by polymerase chain reaction‐restriction fragment length polymorphism (PCR‐RFLP) approach. Primer sequences for *ICAM‐1* K469E and *MKL‐1* −184C/T polymorphisms were designed by Primer Premier 5.0 and synthesized by Sang Biotech (Table 1). The PCR amplifications were performed in a total volume of 10 µL of mixture, containing 0.5 µL forward primer, 0.5 µL reverse primer, 3 µL 10 × Buffer, 1 µL dNTP, 2.5 µL MgCl_2_, 0.5 µL Taq DNA polymerase and 2 µL deionized sterile water. The PCR cycle was as the following: 95°C initial denaturation for 5 minutes; followed by 32 cycles of denaturing at 95°C for 30 seconds, annealing at 60°C for 30 seconds and extension for 30 seconds at 72°C; finally extension at 72°C for 5 minutes.

**Table 1:** Primer sequences of K469E and −184C/T polymorphisms

Locus		Primer sequence
K469E	Forward	5′‐GGAACCCATTGCCCGAGC‐3′
	Reverse	5′‐GGTGAGGATT GCATTAGGTC‐3′
−184C/T	Forward	5′‐GTGCCGTCAGTCACAGGAAGT‐3′
	Reverse	5′‐AAGACTGTCCGCTGGAGAAGTG‐3′

The amplification products were digested by restriction enzyme BstUⅠ at 60°C for 4 hours. Then, digested DNA products were analysed by 3% agarose gel electrophoresis. In *ICAM‐1* K469E polymorphism, only a 223‐bp stripe was observed in KK genotype because it had no restriction enzyme cutting site; three stripes of 223, 136 and 87 bp were observed in KE genotype while two stripes of 136 and 87 bp in EE genotype. As for *MKL‐1* −184C/T polymorphism, the genotyping results were as follows: a stripe of 223 bp for CC genotype, three stripes of 223, 136, 87 bp for CT genotype and two stripes of 136, 87 bp for TT genotype.

### 2.4 Statistical analysis

Data analysis was performed by SPSS.18 statistical software. The genotype and allele frequencies in two groups were compared by χ^2^ test. The correlation of *CAM‐1* and *MKL‐1* gene polymorphisms with CHD was calculated by odds ratios (ORs) and 95% confidence intervals (95% CIs). Multiple logistic regression was adopted for testing the effects of other CHD risk factors. The differences had statistical significance when *P* < .05.

## 3 RESULTS

### 3.1 Characteristics of study subjects

As shown in Table 2, the differences in age, sex and body mass index (BMI) of the two groups had no statistical significance (*P* > .05). But the case group had more smokers and patients with hypertension and diabetes, as well as higher levels of glucose (Glu), serum total cholesterol (TC) and triglyceride (TG) than the control group (*P* < .05). The differences of high‐density lipoprotein cholesterol (HDL‐C) and low‐density lipoprotein cholesterol (LDL‐C) levels between two groups were not statistically significant (*P* > .05).

**Table 2:** General features comparison between cases and controls

Indicator	Case (n = 100)	Control (n = 91)	*P*
Age	59.4 ± 7.33	60.3 ± 6.79	*P* > .05
Sex (male/female)	58/42	53/38	*P* > .05
Smoking	59 (59)	35 (38)	*P* < .05
Body mass index (kg/m^2^)	25.4 ± 3.3	24.7 ± 3.1	*P* > .05
Hypertension	71 (71)	19 (21)	*P* < .05
Diabetes	28 (28)	11 (12)	*P* < .05
Glucose (mmol/L)	6.05 ± 2.79	5.03 ± 0.03	*P* < .05
Total cholesterol (mmol/L)	4.78 ± 1.18	4.31 ± 0.96	*P* < .05
Triglyceride (mmol/L)	2.23 ± 2.17	1.17 ± 0.45	*P* < .05
High density lipoprotein cholesterol (mmol/L)	1.16 ± 0.52	1.21 ± 0.58	*P* > .05
Low density lipoprotein cholesterol (mmol/L)	2.87 ± 0.96	2.78 ± 0.86	*P* > .05

### 3.2 Logistic regression analysis on CHD risk factors

The genotype and allele distributions of *ICAM‐1* K469E and *MKL‐1* −184C/T polymorphisms in case and control groups are shown in Table 3. As shown, the frequencies of these two tested variants did not deviate from the Hardy‐Weinberg equilibrium (HWE), which indicated the representativeness of participants. In the building of Logistic regression, sex, age, smoking status, bodyweight, hypertension, diabetes, levels of Glu, TC, TG, HDL‐C, LDL‐C and polymorphisms of K469E and −184C/T as independent variable, and the existence of CHD as dependent variable. The results of logistic regression analysis are given in Table 4. After adjusting for confounding factors, *ICAM‐1* K469E polymorphism was proved to have no relation with the onset of CHD. While the distribution differences of −184C/T polymorphism TT genotype between case and control groups were of statistical significance (*P* < .05), logistic regression analysis offered a same result. Compared with CC genotype, TT genotype increased 4.92 times the onset risk of CHD (95% CI = 1.32‐26.6). Meanwhile, T allele was closely related to CHD as well (OR = 2.34, 95% CI = 1.34‐5.62). Thus, we confirmed that TT genotype and T allele were susceptible factors for CHD. Other related risk factors like smoking, hypertension, diabetes and TG level were consistent with the recognized CHD risk factors that had been studied before.

**Table 3:** Distributions of K469E and −184C/T polymorphisms in case and control groups

Genotype/Allele	Case (n = 100)	Control (n = 91)	*P*	OR (95% CI)	*P* ^a^	OR^a^ (95% CI)
K469E						
KK	48 (48)	46 (50.5)	—	1.00		
KE	34 (34)	39 (42.9)	.64	0.84 (0.45‐1.54)	.32	0.68 (0.32‐1.45)
EE	18 (18)	6 (6.6)	.04	2.88 (1.05‐7.88)	.07	2.98 (0.93‐9.53)
K	130 (65)	131 (72)	—	1.00		
E	70 (35)	51 (28)	.15	1.38 (0.90‐2.14)	.71	0.87 (0.41‐1.84)
−184C/T						
CC	67 (67)	70 (76.9)	—	1.00		
CT	20 (20)	18 (19.8)	.72	1.61 (0.57‐2.38)	.16	1.88 (0.77‐4.58)
TT	13 (13)	3 (3.3)	.02	4.53(1.24‐16.60)	.02	5.92 (1.32‐26.6)
C	154 (77)	158 (86.8)	—	1.00		
T	46 (23)	24 (13.2)	.02	1.97 (1.15‐3.38)	.04	2.34 (1.34‐5.26)

^a^ Represents adjusted value.

**Table 4:** Logistic regression analysis results of main CHD risk factors

Indicator	*β*	Standard error	*P*	OR	95% CI
Smoking	1.214	.372	.001	3.368	1.624‐6.987
Hypertension	2.423	.403	.000	11.282	5.122‐24.854
Diabetes	1.642	.715	.001	5.496	1.421‐14.32
Triglyceride	1.946	.392	.001	6.716	2.987‐11.46
K469E	1.090	.593	.066	2.975	0.930‐9.517
−184C/T	1.779	.767	.002	5.923	1.318‐26.61

## 4 DISCUSSION

Coronary heart disease is a complex disease. Multiple gene mutations and risk factors lead to the occurrence and development of such disease.^17^ So far, more than 200 risk factors of CHD have been reported, including sex, hypertension, hyperlipidaemia, hyperglycaemia, smoking, obesity and other traditional risk factors.^18^ Hypertension is one of the leading risk factor for CHD. Studies suggest that patients with hypertension (HTN) had 3‐4 times higher risk of suffering from CHD. In the meanwhile, hypertension is independent of other risk factors to have a continuous and rising relationship with the onset of CHD.^19^ Patients with diabetes have high incidence of CVD, and their risk of developing CHD is 2‐4 times as high as that of non‐diabetic patients.^20^, ^21^ Likewise, smoking is an independent risk factor for CHD. Smokers have been certified to have 1.5‐4 times higher risk of being afflicted with CHD than non‐smokers. Furthermore, people who begin smoking at an early age are at a higher risk of CHD.^22^

With the deepening of the researches and development of biological technology, a growing body of evidence has confirmed the genetic susceptibility for CHD, and multiple genes have been found to be closely associated with the susceptibility for CHD. Hinohara et al firstly ascertained the correlation of *MKL‐1* −184C/T polymorphism with the onset of CHD in 2009, and the association was replicated in both Japanese (OR = 1.25, 95%CI = 1.04‐1.49) and Korean (OR = 1.26, 95% CI = 1.01‐1.58) populations.^23^ Other reports have demonstrated that the high expression of *MKL‐1* might play important roles in the occurrence and development of atherosclerosis.^15^, ^24^ The present study discovered that −184C/T SNP of *MKL‐1* gene was closely related to the onset of CHD, and TT genotype increased the onset risk of such disease. T allele frequency was significantly higher in case group than that in control group, and it was suggested to be a risk factor for CHD. The distributions of TT genotype and T allele in case and control groups still had statically significant differences after logistic regression analysis, which attested the correlation with the onset of CHD. This is consistent with a previous study, and they also found that the ‐ 184C > T polymorphism of MKL1 is an important risk factor for CHD in Han nationality of Henan Province. The homozygosity of T allele is related to the risk of CHD and the severity of stenosis.^25^

Many researches on the relationship between *ICAM‐1* polymorphisms and inflammatory diseases have been reported.^26^, ^27^ Correlation analyses on *ICAM‐1* K469E polymorphism and CHD have been reported as well both in domestic and abroad,^28^, ^29^, ^30^ but the results are inconclusive and contradictory. Liu et al conducted meta‐analysis and found that rs5498 polymorphism was related to the reduction of CAD risk in Caucasians, but not Asians, which may be caused by different races.^31^ The results of our study illustrated that after being assessed by logistic regression analysis, *ICAM‐1* K469E polymorphism was found to have no obvious association with CHD in the Chinese population.

Some limitations of our research cannot be ignored. First of all, in the case‐control study, our sample is relatively small, it is necessary to expand the sample size to further verify our results. Secondly, our research objects are all Han nationality, the possibility of different genetic background and surroundings caused by ethnic and regional differences might affect experimental results. Therefore, a lager or different population should be taken into account to confirm the results.

In summary, the present study suggested the correlation between *MKL‐1* −184C/T polymorphism and CHD, and T allele might be a susceptible factor for the disease. Meanwhile, the coexistence of TT genotype with smoking status, hypertension, diabetes or high TG level would significantly increase the morbidity of CHD.

## CONFLICT OF INTEREST

None.

## AUTHOR CONTRIBUTIONS

**Cungang Wu:** Conceptualization (equal); Data curation (equal); Formal analysis (equal); Writing–review & editing (equal). **Chao Huang:** Methodology (equal); Resources (equal); Software (equal); Writing–original draft (equal).

## ETHICAL APPROVAL

With the approval of The First Affiliated Hospital of Jinzhou Medical University Ethics Committee, written informed consent was obtaining from every subject.

## Data Availability

All data generated or analysed during this study are included in this article.
